# Improving the accuracy of two-sample summary-data Mendelian randomization: moving beyond the NOME assumption

**DOI:** 10.1093/ije/dyy258

**Published:** 2018-12-18

**Authors:** Jack Bowden, Fabiola Del Greco M, Cosetta Minelli, Qingyuan Zhao, Debbie A Lawlor, Nuala A Sheehan, John Thompson, George Davey Smith

**Affiliations:** 1 MRC Integrative Epidemiology Unit, University of Bristol, Bristol, UK; 2 Population Health Sciences, University of Bristol, Bristol, UK; 3 Institute for Biomedicine, Eurac Research, Bolzano, Italy; 4 Population Health and Occupational Disease, NHLI, Imperial College, London, UK; 5 Department of Statistics, The Wharton School, University of Pennsylvania, Philadelphia, PA, USA; 6 Department of Health Sciences, University of Leicester, Leicester, UK

**Keywords:** Two-sample summary-data Mendelian randomization, inverse-variance weighted estimate, Cochran’s *Q* statistic, outlier detection

## Abstract

**Background:**

Two-sample summary-data Mendelian randomization (MR) incorporating multiple genetic variants within a meta-analysis framework is a popular technique for assessing causality in epidemiology. If all genetic variants satisfy the instrumental variable (IV) and necessary modelling assumptions, then their individual ratio estimates of causal effect should be homogeneous. Observed heterogeneity signals that one or more of these assumptions could have been violated.

**Methods:**

Causal estimation and heterogeneity assessment in MR require an approximation for the variance, or equivalently the inverse-variance weight, of each ratio estimate. We show that the most popular ‘first-order’ weights can lead to an inflation in the chances of detecting heterogeneity when in fact it is not present. Conversely, ostensibly more accurate ‘second-order’ weights can dramatically increase the chances of failing to detect heterogeneity when it is truly present. We derive modified weights to mitigate both of these adverse effects.

**Results:**

Using Monte Carlo simulations, we show that the modified weights outperform first- and second-order weights in terms of heterogeneity quantification. Modified weights are also shown to remove the phenomenon of regression dilution bias in MR estimates obtained from weak instruments, unlike those obtained using first- and second-order weights. However, with small numbers of weak instruments, this comes at the cost of a reduction in estimate precision and power to detect a causal effect compared with first-order weighting. Moreover, first-order weights always furnish unbiased estimates and preserve the type I error rate under the causal null. We illustrate the utility of the new method using data from a recent two-sample summary-data MR analysis to assess the causal role of systolic blood pressure on coronary heart disease risk.

**Conclusions:**

We propose the use of modified weights within two-sample summary-data MR studies for accurately quantifying heterogeneity and detecting outliers in the presence of weak instruments. Modified weights also have an important role to play in terms of causal estimation (in tandem with first-order weights) but further research is required to understand their strengths and weaknesses in specific settings.

**Key Messages**

- Two-sample summary-data Mendelian randomization requires the specification of inverse-variance weights for model fitting, heterogeneity quantification and outlier detection amongst a set of causal estimates.
- Heterogeneity indicates a possible violation of the necessary IV or modelling assumptions of which pleiotropy is a likely major cause.
- First-order weights can inflate the type I error rate of Cochran’s *Q* statistic for detecting heterogeneity about the inverse-variance weighted (IVW) estimate when the NO Measurement Error (NOME) assumption is strongly violated (as judged by a low *F*-statistic) and the true causal effect of interest is non-zero.
- Second-order weights can reduce the power of Cochran’s *Q* statistic for detecting heterogeneity about the IVW estimate when the NOME assumption is violated.
- Modified weights (developed in this paper) preserve the type I error rate of Cochran’s *Q* statistic, whilst maintaining its statistical power.
- ‘Exact’ modified weights should be used for global tests of heterogeneity. ‘Iterative’ modified weights should be used to assess the outlier status of individual single-nucleotide polymorphisms (SNPs).
- IVW estimates obtained using exact weights are naturally corrected for regression dilution bias, and work well with large numbers of instruments, but can be imprecise relative to other weighting schemes with small numbers of weak instruments.
- Regardless of the number or strength of instruments used, first-order weights always furnish unbiased IVW estimates and preserve the type I error rate under the causal null.

## Introduction

Mendelian randomization (MR)^1^ is an instrumental variable approach that uses genetic data, typically in the form of single-nucleotide polymorphisms (SNPs), to assess whether a modifiable exposure exerts a causal effect on a health outcome in the presence of unmeasured confounding. A particular MR study design gaining in popularity instead combines publically available summary data on SNP–exposure and SNP–outcome associations from two separate studies for large numbers of uncorrelated variants within the framework of a meta-analysis. These studies should contain no overlapping individuals (to ensure independence) but should also originate from the same source population. This is referred to as two-sample summary-data MR.^2^ Providing the necessary modelling assumptions are met and the chosen set of SNPs are all valid instrumental variables, an inverse-variance weighted (IVW) average of their individual causal ratio estimates provides an efficient and consistent estimate for the causal effect. This is referred to as the IVW estimate (see Box 1). Cochran’s *Q* statistic, which is derived from the IVW estimate, should follow a $\chi^{2}$ distribution with degrees of freedom equal to the number of SNPs minus 1. Excessive heterogeneity is an indication that either the modelling assumptions have been violated, or that some of the genetic variants violate the IV assumptions—e.g. by exerting a direct effect on the outcome not through the exposure.^3^ This is termed ’horizontal pleiotropy’.^4^^,^^5^ For brevity, we will refer to problematic horizontal pleiotropy simply as pleiotropy from now on.

The presence of heterogeneity due to pleiotropy does not necessarily invalidate an MR study. If across all variants (i) the amount of pleiotropy is independent of instrument strength (the InSIDE assumption^6^) and (ii) it has a zero mean, then a standard random-effects meta-analysis will still yield reliable inferences.^6^^,^^7^ Although many MR methods now exist that offer robustness to pleiotropy, in this paper, we focus solely on the standard IVW estimate.

### Choice of weights in two-sample summary-data MR

Typically, ‘first-order’ inverse-variance weights are used to calculate both the IVW estimate and Cochran’s *Q*. First-order weights ignore uncertainty in the denominator of the ratio estimate, which is equivalent to making the ‘NO Measurement Error’ (NOME) assumption, as defined in Refs.^7^^,^^8^ This nomenclature is chosen to remind the practitioner that the SNP–exposure association estimates are only equal to the true associations when measured with infinite precision (or without error). The NOME assumption does not relate to absence of measurement error in the exposure itself, which can also be problematic for MR studies.^9^ Although the NOME assumption is never completely satisfied, strong violation (via the use of weak genetic instruments) induces classical regression dilution bias in the IVW estimate towards the null. So-called ‘second-order’ weights attempt to better acknowledge the full uncertainty in the ratio estimate of causal effect from each SNP^10^^,^^11^ (see Box 1). It may appear obvious that second-order weights should be used as standard within an MR study to calculate the IVW estimate and Cochran’s *Q*. In fact, Thompson *et al*.^12^ showed that second-order weighting produces causal estimates that are generally more biased than first-order weighting. The ability of first- and second-order weighting to furnish reliable *Q* statistics has yet to be fully explored.

## Methods

It is possible to view Cochran’s *Q* statistic not just as a method for quantifying heterogeneity, but as a tool for directly estimating the causal effect. That is, the IVW estimate actually minimizes Cochran’s *Q*. We use this fact to derive a generalized estimating equation based on an extended version of Cochran’s *Q* statistic (see Box 2), where its weight term is allowed to be a function of the causal-effect parameter. We show that first-order and second-order weighting are special cases of this general weight function. Using this formulation, we propose two new procedures for causal-effect estimation and heterogeneity quantification.

Our first procedure is termed the ‘iterative’ approach. It iteratively updates the weight term with improved guesses for the causal parameter, using the first-order IVW estimate as a starting point. This procedure is closely related to the ‘two-step generalized method of moments (GMM)’ estimator^13^ used in econometrics. Our contribution has been to describe how it can be implemented using Cochran’s *Q* statistic in the two-sample summary-data MR setting. It will be shown that the iterative IVW approach improves causal-effect estimation and heterogeneity detection compared with first- and second-order weighting. However, regardless of the number of iterations performed, this procedure will not in general yield the same results as that obtained from directly minimizing Cochran’s *Q*, where the weight term is allowed to be a proper function of the causal-effect parameter *β*. We refer to this second procedure as the ‘exact’ approach. The exact IVW estimate can be viewed as analogous to the limited-information maximum-likelihood (LIML) estimate, translated to the two-sample summary-data MR setting.^14^ For further details, see Box 2.

### Estimation and inference after detection of pleiotropy

Box 2 describes how to use *Q* statistics to calculate the IVW estimate under a fixed-effect model and to test for the presence of heterogeneity due to pleiotropy. If substantial heterogeneity is detected, inferences about the causal effect need to be adjusted to take this additional uncertainty into account, by assuming a random-effects model.^15^^,^^16^ In Supplementary Appendix 1 (available as Supplementary data at *IJE* online), we describe in detail how to generalize the *Q* statistics to obtain point estimates, standard errors and confidence intervals for the first-order, second-order, iterative and exact IVW estimate under both fixed and random-effects models (the multiplicative model is currently preferred for MR studies). This task is straightforward for the first-order, second-order and iterative weighting approaches because they can be fitted using standard regression software. Bespoke methods are needed for exact weighting, however, and a short summary of this particular approach is provided in Box 3. Specifically, in the fixed-effect case, we describe how to invert the exact *Q* statistic to get a 95% confidence interval for the exact weighted IVW estimate. In the random-effects case, we describe how to jointly estimate the causal effect and multiplicative over-dispersion parameter using a system of two estimating equations. A non-parametric bootstrap algorithm is then proposed to obtain a confidence interval for the causal effect.

### Performance of the *Q* statistics under no pleiotropy

We now assess the extent to which *Q* statistics derived using first-order, second-order, iterative and exact weighting erroneously detect heterogeneity due to pleiotropy when it is not present (i.e. its type I error rate). To assess this, two-sample summary-data MR studies comprising 25 SNP–exposure and SNP–outcome association estimates were generated from models with no heterogeneity due to pleiotropy. This furnished a set of ratio estimates between which no additional variation should exist as their instrument strength grows large (because NOME is satisfied) or if the causal effect (*β*) equals zero. To highlight this, we simulated MR studies with a range of instrument strengths—from weak (a mean *F*-statistic of 10) to strong (a mean *F*-statistic of 100). Further details of the simulation study set up are described in Supplementary Appendix 2 (available as Supplementary data at *IJE* online).

Table 1 (columns 2–9) show the mean *Q* statistic and the probability of the *Q* statistic detecting heterogeneity at the 5% significance level (the type I error rate), when using first-order, second-order, iterative and exact weights. Five different mean *F*-statistic values were considered for *β *= 0 (no causal effect), *β *= 0.05 and *β *= 0.1, giving 15 scenarios in total. Four iterations were used for the iterative weighting method, as this was sufficient to ensure convergence. We note that, in the absence of a causal effect (*β *= 0), first-order weights are exactly correct. Furthermore, in the presence of a causal effect, when the mean *F*-statistic is 100, all weighting methods are near-exact. Under the causal null, all weighting schemes control the type I error rate for detecting heterogeneity. Second-order weighting is extremely conservative in this respect with weak instruments, however (e.g. a type I error rate near zero when *F *=* *10).

**Table 1.** Mean *Q* statistic and type I error rate (T1E) of first-order, second-order, iterative (four iterations were performed) and exact weighting

Mean	First-order *w_j_*		Second-order *w_j_*		Modified *w_j_*			
					Iterative		Exact	
*F*	*Q*	T1E(*Q*)	*Q*	T1E(*Q*)	*Q*	T1E(*Q*)	*Q*	T1E(*Q*)
No heterogeneity, *β* = 0								
100	23.9	0.044	22.8	0.022	23.9	0.044	23.9	0.044
61	24.1	0.052	21.9	0.016	24.1	0.051	24.1	0.051
40	23.9	0.049	20.3	0.006	23.9	0.048	23.9	0.048
25	24.0	0.052	17.7	0.002	23.9	0.051	23.9	0.051
10	24.0	0.052	12.3	0.000	23.6	0.047	23.4	0.042
No heterogeneity, *β* = 0.05								
100	24.2	0.053	22.9	0.028	24.0	0.049	24.0	0.049
61	24.4	0.058	21.9	0.017	24.0	0.051	24.0	0.051
40	24.7	0.064	20.3	0.007	23.9	0.050	23.9	0.049
25	25.9	0.092	17.8	0.002	24.1	0.052	23.9	0.048
10	31.4	0.272	13.4	0.000	25.6	0.095	23.7	0.043
No heterogeneity, *β* = 0.1								
100	24.7	0.065	22.8	0.027	23.9	0.052	23.9	0.051
61	25.6	0.084	21.8	0.017	23.9	0.048	23.9	0.047
40	27.3	0.132	20.5	0.009	24.1	0.053	24.0	0.050
25	31.7	0.282	18.2	0.003	24.4	0.060	23.9	0.048
10	53.9	0.792	15.8	0.004	27.8	0.166	23.9	0.051

Results are the average of 10 000 simulated data sets. Type I error rate (T1E(Q)) refers to the proportion of times *Q* is greater than the upper 95th percentile of a $\chi_{24}^{2}$ distribution.

In the presence of a causal effect, first-order weights underestimate the true variability amongst the ratio estimates as the mean *F*-statistic reduces. The associated *Q* statistics are then too large on average (i.e. positively biased beyond their expected value of 24). This inflates the type I error rate for detecting pleiotropy beyond nominal levels (e.g. a type I error rate of ≈80% when *F *=* *10 and *β *= 0.1). Second-order weighting continues to over-correct the *Q* statistic so that it is negatively biased, thereby removing *any* ability to detect heterogeneity at all. In contrast, iterative weights are much more effective at preserving the type I error rate of the *Q* statistic at its nominal level, unless the mean *F*-statistic is very low (indicating weak instruments). Exact weighting perfectly controls the type I error rate of Cochran’s *Q* across all the scenarios considered. Supplementary Appendix 2 (available as Supplementary data at *IJE* online) shows equivalent results for MR studies of 10 and 100 variants, with highly similar results.

**Figure 1.**
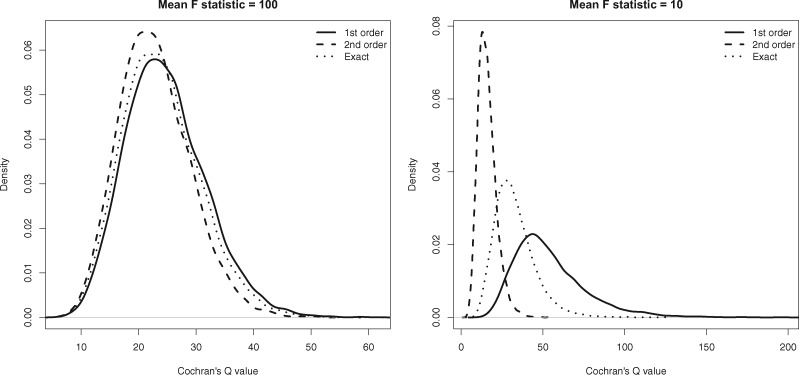
Distribution of *Q* statistics (with 25 degrees of freedom) using first-order, second-order and exact weights. The causal effect β = 0.1 and the mean *F*-statistic equals 100 (left) and 10 (right) respectively.

Figure 1 (left and right) shows the distribution of *Q* statistics using first-order, second-order and exact weights for *β *= 0.1 and when the mean *F*-statistic is 100 and 10. This illustrates how exact weighting ensures Cochran’s *Q* statistic is faithful to its correct null distribution.

### Power to detect pleiotropy

**Figure 2.**
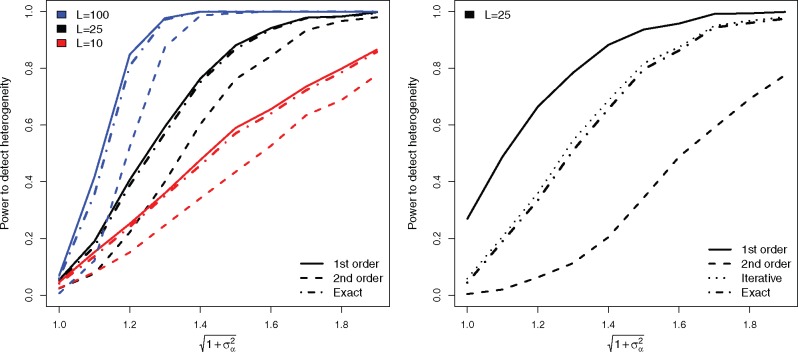
Left: Power of Cochran’s *Q* statistic to detect heterogeneity as a function of the pleiotropy variance and number of SNPs (*L*) using first-order, second-order and exact weights. Pleiotropy is simulated under a multiplicative random-effects model. The causal effect is equal to 0.05 and the mean *F*-statistic is 61. Top group: L=100; middle group: L=25; bottom group: L=10. Right: Equivalent power plot except the causal effect is equal to 0.1 and the mean *F*-statistic is 25.

In Table 1, the type I error rate of Cochran’s *Q* statistic for detecting heterogeneity using second-order weights was below its nominal level. This is detrimental if it translates into a low statistical power to detect heterogeneity when it *is* truly present. Figure 2 (left) shows the power of Cochran’s *Q* to detect heterogeneity at the 5% significance level as a function of first-order, second-order, iterative and exact weights when data are simulated under a multiplicative random-effects model with heterogeneity due to pleiotropy of increasing magnitude [specifically, Equation (2) in Supplementary Appendix 1 (available as Supplementary data at *IJE* online) was used].

The simulation is repeated for MR analyses with 10, 25 and 100 SNPs. For all simulations, the causal effect equalled 0.05 and the mean *F*-statistic equalled 61. We see that the power of Cochran’s *Q* to detect heterogeneity approaches 100% for all weighting schemes as the pleiotropy variance increases. Power also increases with the number of SNPs. The power of iterative or exact weights is near identical, so we only show results for the exact weights for clarity. The most striking result in this plot is that the power of second-order weighting always lags considerably behind that of first-order or exact weights.

Figure 2 (right) shows the results of a near identical simulation for the case *L *=* *25, except that the causal effect is set to 0.1 and the mean *F*-statistic is equal to 25. We see that the power to detect heterogeneity is always greatest when using first-order weights, but only because its power curve starts at a baseline level of 28% when there is no pleiotropy. This corresponds to the type I error rate observed in row 14 of Table 1. The power of iterative and exact weighting starts at the correct 5% level and rapidly increases to 100% as the pleiotropy variance increases. The two implementations of our modified weights can be differentiated in this simulation, with the iterative approach being slightly more powerful. The power of second-order weighting, unsurprisingly, lags considerably behind the rest. Equivalent plots for data simulated under an additive pleiotropy model are shown in Supplementary Appendix 3 (available as Supplementary data at *IJE* online) and are highly similar.

### Detecting outliers using individual components of *Q*

When heterogeneity is detected by the IVW model, it is interesting to investigate whether this is contributed to by all SNPs or whether instead a small number of SNPs are responsible. Under the null hypothesis of no heterogeneity, *Q* should follow an appropriate $\chi_{L-1}^{2}$ distribution, with *L* being the number of SNPs. Likewise, each individual component of *Q* can be approximated by a $\chi_{1}^{2}$ distribution. If an individual SNP’s *Q* contribution is extreme (e.g. above the 5% threshold of 3.84 or instead a Bonferroni-corrected threshold), then it may be desirable to exclude the SNP in a sensitivity analysis. Although we do not want to advocate a rigid, blanket policy of outlier removal, in Supplementary Appendix 4 (available as Supplementary data at *IJE* online), we illustrate via simulation how the reliability of such a procedure depends on the choice of weights. The simulation (with 26 SNPs and a single larger outlier) is motivated by the real data example in the following section. In this instance, our simulation suggests that iterative rather than exact weights are best at correctly identifying outliers due to pleiotropy.

### Estimator performance with and without pleiotropy

Table 2 shows the performance of the first-order, second-order, iterative and exact weigthing in providing accurate point estimates, standard errors and confidence intervals for the causal effect under a fixed-effect (no heterogeneity) model for MR analyses of 25 variants. For exact weighting, we show the empirical coverage using two different methods: a symmetric 95% confidence interval (labelled ‘CF_1_’) and a 95% confidence interval obtained from inverting its *Q* statistic (labelled ‘CF_2_’), as described in Box 3. Importantly, all methods give reliable unbiased estimates with correct coverages under the causal null hypothesis. In the presence of a non-zero causal effect, first-order and second-order IVW estimates are increasingly affected by regression dilution bias (and consequently worsening coverage) as the instrument strength decreases. Iterative weights also produce IVW estimates that suffer from regression dilution bias and sub-optimal coverage, but to a lesser extent than first- or second-order weighting. Exact weighting perfectly removes the effect of regression dilution bias (although the precision of the estimate is reduced) and confidence intervals obtained via the inversion method have the correct coverage. Equivalent results for MR studies with 10 and 100 SNPs are shown in Supplementary Appendix 5 (available as Supplementary data at *IJE* online). When only 10 SNPs are available and they are all weak, the coverage of the inverted confidence interval for the exact IVW estimate is slightly conservative (e.g. 96–98% instead of 95%). As the number of SNPs increases to 100, coverage is very close to the nominal 95% level irrespective of instrument strength.

**Table 2.** Mean causal estimate ${\hat{\beta }}_{IVW}$, standard error (SE) and coverage frequency (CF) of the 95% confidence interval when using first-order, second-order, iterative and exact weights

Mean	First-order *w_j_*	Second-order *w_j_*	Modified *w_j_*		
			Iterative	Exact	
*F*	${\hat{\beta }}_{IVW}$(SE); CF	${\hat{\beta }}_{IVW}$(SE); CF	${\hat{\beta }}_{IVW}$(SE); CF	${\hat{\beta }}_{IVW}$(SE); CF_1_	CF_2_
No heterogeneity, *β* = 0					
100	0.000 (0.011); 0.952	0.000 (0.011); 0.951	0.000 (0.011); 0.952	0.000 (0.011); 0.961	0.948
61	0.000 (0.011); 0.947	0.000 (0.011); 0.947	0.000 (0.011); 0.948	0.000 (0.011); 0.956	0.946
40	0.000 (0.011); 0.954	0.000 (0.010); 0.952	0.000 (0.011); 0.955	0.000 (0.011); 0.957	0.946
25	0.000 (0.011); 0.947	0.000 (0.010); 0.941	0.000 (0.011); 0.949	0.000 (0.011); 0.942	0.949
10	0.000 (0.009); 0.952	0.000 (0.007); 0.928	0.000 (0.009); 0.958	0.000 (0.010); 0.836	0.958
No heterogeneity, *β* = 0.05					
100	0.049 (0.011); 0.952	0.049 (0.011); 0.951	0.049 (0.011); 0.954	0.050 (0.011); 0.962	0.952
61	0.049 (0.011); 0.948	0.047 (0.011); 0.944	0.049 (0.011); 0.952	0.050 (0.011); 0.961	0.953
40	0.048 (0.011); 0.939	0.045 (0.011); 0.918	0.048 (0.011); 0.943	0.050 (0.012); 0.951	0.946
25	0.046 (0.011); 0.910	0.041 (0.010); 0.819	0.046 (0.011); 0.923	0.050 (0.012); 0.940	0.954
10	0.033 (0.010); 0.589	0.027 (0.008); 0.286	0.034 (0.011); 0.670	0.051 (0.012); 0.868	0.957
No heterogeneity, *β* = 0.1					
100	0.099 (0.011); 0.945	0.097 (0.011); 0.945	0.099 (0.012); 0.950	0.100 (0.012); 0.963	0.946
61	0.098 (0.011); 0.932	0.095 (0.011); 0.920	0.098 (0.012); 0.944	0.100 (0.012); 0.956	0.947
40	0.097 (0.012); 0.911	0.091 (0.011); 0.859	0.097 (0.012); 0.933	0.100 (0.013); 0.954	0.951
25	0.092 (0.012); 0.844	0.083 (0.011); 0.649	0.092 (0.013); 0.896	0.101 (0.014); 0.947	0.955
10	0.065 (0.013); 0.348	0.055 (0.010); 0.094	0.072 (0.014); 0.518	0.102 (0.016); 0.895	0.964

Number of variants *L* = 25. CF_1_ = coverage of a symmetric 95% confidence interval, CF_2_ = coverage of inverted *Q* statistic confidence interval.

Table 3 shows equivalent results when summary-data sets of 25 SNPs are simulated under a multiplicative random-effects model allowing for pleiotropy. The data are simulated so that the variability of the ratio estimates is twice that expected in the absence of pleiotropy (i.e. the variance inflation parameter ϕ = 2). The performance of each approach follows a similar pattern to that presented for the fixed-effect case in Table 2, with first-order, second-order and iterative weights adversely affected by weak instrument bias and under coverage. The exact IVW estimate and its corresponding variance inflation parameter estimate are approximately unbiased. The non-parametric bootstrap procedure yields confidence intervals with approximately correct coverage. As before, confidence intervals have a tendency to be slightly conservative when the instruments are weak. Equivalent results for MR studies with 10 and 100 SNPs are shown in Supplementary Appendix 6 (available as Supplementary data at *IJE* online). As the number of SNPs increases, the coverage of the exact IVW estimate’s confidence interval is increasingly closer to the nominal level.

**Table 3.** Mean causal estimate ${\hat{\beta }}_{IVW}$, standard error (SE) and coverage frequency (CF) of the 95% confidence interval when using first-order, second-order, iterative and exact weights

Mean	First-order *w_j_*	Second-order *w_j_*	Modified *w_j_*		
			Iterative	Exact	
*F*	${\hat{\beta }}_{IVW}$(SE); CF	${\hat{\beta }}_{IVW}$(SE); CF	${\hat{\beta }}_{IVW}$(SE); CF	${\hat{\beta }}_{IVW}$(SE); CF	$\hat{\phi }$
Heterogeneity, *β* = 0					
100	0.000(0.016); 0.949	0.000 (0.015); 0.950	0.000 (0.016); 0.950	0.000 (0.016); 0.939	2.000
61	0.000 (0.016); 0.950	0.000 (0.015); 0.951	0.000 (0.016); 0.951	0.000 (0.016); 0.940	2.004
40	0.000 (0.016); 0.953	0.000 (0.014); 0.951	0.000 (0.016); 0.955	0.000 (0.016); 0.944	1.999
25	0.000 (0.015); 0.949	0.000 (0.013); 0.945	0.000 (0.015); 0.954	0.000 (0.017); 0.945	2.003
10	0.000 (0.013); 0.952	0.000 (0.009); 0.924	0.000 (0.013); 0.960	0.000 (0.037); 0.970	1.943
Heterogeneity, *β* = 0.05					
100	0.050 (0.016); 0.948	0.048 (0.015); 0.947	0.050 (0.016); 0.949	0.050 (0.016); 0.938	2.002
62	0.049 (0.016); 0.951	0.046 (0.015); 0.943	0.049 (0.016); 0.954	0.050 (0.016); 0.943	1.998
40	0.048 (0.016); 0.949	0.044 (0.014); 0.924	0.048 (0.016); 0.953	0.050 (0.017); 0.943	1.995
25	0.046 (0.015); 0.933	0.039 (0.013); 0.839	0.046 (0.016); 0.940	0.051 (0.018); 0.944	1.987
10	0.033 (0.014); 0.719	0.025 (0.010); 0.378	0.034 (0.015); 0.778	0.051 (0.037); 0.960	1.967
Heterogeneity, *β* = 0.1					
100	0.099 (0.016); 0.947	0.096 (0.016); 0.942	0.099 (0.016); 0.952	0.100 (0.016); 0.942	2.005
61	0.098 (0.016); 0.941	0.092 (0.016); 0.922	0.098 (0.017); 0.951	0.100 (0.017); 0.941	2.004
40	0.097 (0.016); 0.932	0.088 (0.015); 0.862	0.097 (0.017); 0.947	0.101 (0.017); 0.940	2.003
25	0.092 (0.016); 0.888	0.078 (0.015); 0.676	0.093 (0.018); 0.924	0.101 (0.019); 0.942	2.003
10	0.065 (0.016); 0.456	0.051 (0.012); 0.131	0.072 (0.018); 0.639	0.101 (0.042); 0.956	2.023

*L* = 25. $\hat{\phi }$ equals the variance inflation factor estimate (true value = 2).

### Power to detect a causal effect

In Supplementary Appendix 7 (available as Supplementary data at *IJE* online), we show the power of first-order, second-order, iterative and exact weighting to detect a causal effect for MR studies of 10, 25 and 100 SNPs when the data are generated from the same multiplicative random-effects model. These simulations highlight a downside of exact weighting for causal estimation: when there are only a small number of weak instruments, its power can be considerably lower. For example, when *F *=* *10 and the causal effect is 0.05, its power is just under half that of the first-order IVW estimate (29 vs 13%). However, the power difference reduces considerably for 25 SNPs (e.g. 60 vs 40%) and is effectively zero for 100 SNPs. The power of iterative weighting is much more comparable to that of first-order weighting, but always slightly lower.

## Applied example

**Figure 3.**
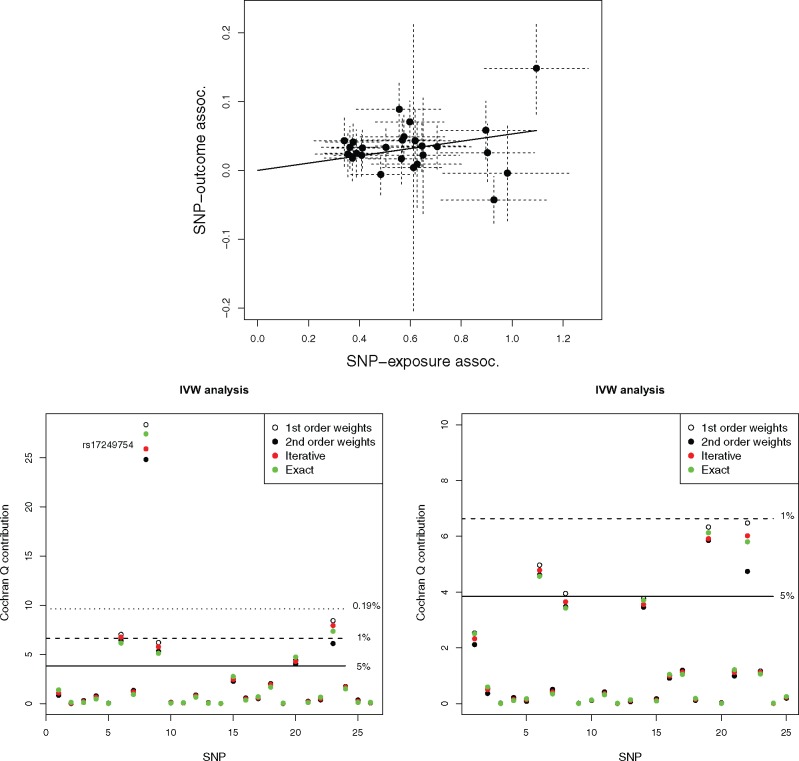
Top: Scatter plot of SNP–outcome associations ${\hat{\Gamma }}_{j}$ vs SNP–exposure associations ${\hat{\gamma }}_{j}$. IVW estimate shown as a black slope. Bottom-left: *Q* contribution plots for the same data. Bottom-right: *Q* contributions after removal of rs17249754.

Figure 3 (top) shows a scatter plot of summary-data estimates for the associations of 26 genetic variants with systolic blood pressure (SBP, the exposure) and coronary heart disease (CHD, the outcome). SNP–exposure association estimates were obtained from the International Consortium for Blood Pressure consortium (ICBP).^17^ SNP–CHD association odds ratios were collected from Coronary ARtery Disease Genome-Wide Replication And Meta-Analysis (CARDIoGRAM) consortium,^18^ which are plotted (and subsequently modelled) on the log-odds ratio scale by making a normal approximation. These data have previously been used in a two-sample summary-data MR analysis by Ference *et al*.^19^ and Lawlor *et al*.,^20^ but we extend their original analysis here by applying our modified weights and conducting a more in-depth inspection of each variant’s contribution to the overall heterogeneity. The mean *F*-statistic for these data is 61. Using first-order weights, the IVW estimate, which represents the causal effect of a 1-mmHg increase in SBP on the log-odds ratio of CHD, is 0.053. This is shown as the slope of a solid black line passing through the origin. Cochran’s *Q* statistic based on first-order weights is equal to 67.1, indicating the presence of substantial heterogeneity. For this reason, only random-effects models were used to derive point estimates, confidence intervals and *p*-values for the causal effect.

Table 4 shows the results of further IVW analyses using all weighting schemes. All schemes detect significant heterogeneity. As expected, the observed heterogeneity is largest when using first-order weights, smallest when using second-order weights and in between the two when using modified weights. Point estimates and standard errors are in good agreement across the different weights, because the mean instrument strength is high. Exact weighting gives the largest point estimate 0.054 under a random-effects model. This is followed by first-order and then second-order weights, respectively. This ordering is as expected, given their relative susceptibility to regression dilution bias.

**Table 4.** IVW and weighted median analyses of the causal effect of SBP on CHD risk for the complete data (top) and with SNP rs17249754 removed (bottom)

Method (weights)	Estimate (CI)	SE	*P*-value	Het. Stat (*p*)	$\hat{\phi }$
All 26 SNPs					
Causal estimate					
IVW (first—RE)	${\hat{\beta }}_{IVW}$: 0.053 (0.032, 0.075)	0.010	3.01 × 10^–5^	*Q* = 67.1 (1.03 × 10^–5^)	2.68
IVW (second—RE)	${\hat{\beta }}_{IVW}$: 0.050 (0.029, 0.071)	0.010	4.54 × 10^–5^	*Q* = 58.8 (1.54 × 10^–4^)	2.35
IVW (iterative—RE)	${\hat{\beta }}_{IVW}$: 0.054 (0.032, 0.075)	0.010	2.40 × 10^–5^	*Q* = 62.7 (4.43 × 10^–5^)	2.51
IVW (exact—RE)	${\hat{\beta }}_{IVW}$: 0.054 (0.027, 0.082)	0.014	4.60 × 10^–4^	*Q* = 62.4 (4.84 × 10^–5^)	2.61
Weighted median (first-order weights)					
Weighted median	${\hat{\beta }}_{WM}$: 0.063 (0.042, 0.084)	0.011	4.90 × 10^–6^	–	–
SNP rs17249754 removed					
Causal estimate					
IVW (first—RE)	${\hat{\beta }}_{IVW}$: 0.066 (0.049, 0.083)	0.008	2.63 × 10^–8^	*Q* = 35.0 (0.068)	1.46
IVW (second—RE)	${\hat{\beta }}_{IVW}$: 0.063 (0.047, 0.080)	0.008	4.06 × 10^–8^	*Q* = 30.6 (0.164)	1.27
IVW (iterative—RE)	${\hat{\beta }}_{IVW}$: 0.066 (0.049, 0.083)	0.008	2.90 × 10^–8^	*Q* = 32.8 (0.107)	1.37
IVW (exact—RE)	${\hat{\beta }}_{IVW}$: 0.067 (0.049, 0.085)	0.009	8.37 × 10^–8^	*Q* = 32.8 (0.108)	1.39
Weighted median (first-order weights)					
Weighted median	${\hat{\beta }}_{WM}$: 0.065 (0.044, 0.087)	0.011	2.33 × 10^–6^	–	

${\hat{\beta }}_{IVW}$ is the IVW estimate. ${\hat{\beta }}_{WM}$ is the weighted median estimate. All IVW estimates fitted under a multiplicative random-effects model (RE), where $\hat{\phi }$ refers to the variance inflation factor estimate. The weighted median naturally accounts for heterogeneity via a bootstrapped variance.

For comparison, we also report the weighted median,^21^${\hat{\beta }}_{WM}$, that can identify the causal effect when up to (but not including) half of the information in the analysis stems from genetic variants that are invalid IVs. Its estimate, which is calculated using first-order weights, is 0.063. Although all approaches provide strong evidence in favour of a non-zero causal effect, the exact random-effects IVW estimate is the least precise of all estimates. Consequently, its *p*-value for testing the causal null hypothesis is the largest of all.

Figure 3 (bottom-left) shows the individual contribution to Cochran’s *Q* statistic under each weighting scheme. Horizontal lines have been drawn to indicate the location of the 5th, 1st and 0.19th percentiles of a $\chi_{1}^{2}$ in order to help assess the magnitude of the contributions. The 0.19th percentile is derived as a 0.05 threshold adjusted for multiple testing using the Bonferroni correction. We see that the eighth SNP in our list (rs17249754) is responsible for the vast majority of the excess heterogeneity. Its contribution, *Q*_8_, ranges from approximately 24.5 to 28, depending on weighting. Variant rs17249754 sits in the ATPase plasma membrane Ca2+ transporting 1 (*ATP2B1*) gene, which is involved in intracellular calcium homeostasis, and is strongly associated with higher SBP. However, in the CARDIoGRAM consortium, it is associated with reduced risk of CHD.

Since rs17249754 is also a strong instrument and is potentially pleiotropic, its presence in the data could lead to the InSIDE assumption being violated. We therefore opt to remove it in a further sensitivity analysis and Table 4 show the results. All IVW estimates increase by around 20% (lying between 0.063 and 0.067) but are ordered as before. Removal of rs17249754 leads to a dramatic reduction in the amount of heterogeneity present in the data, as referenced by *Q* statistics between 30 and 35 for all methods. Figure 3 (bottom-right) shows the updated contributions of each SNP to the various *Q* statistics after removing rs17249754. If only first-order weighting were available, it might be tempting to exclude further variants from the analysis, but this signal is appropriately tempered when using exact weights. The weighted median estimate without rs17249754 is 0.065 (compared with 0.063 with). This highlights its inherent robustness to outliers, which is a major strength.

## Discussion

In this paper, we have demonstrated the limitations of first- and second-order weighting when used for IVW analysis in two-sample summary-data MR. Most importantly, we highlight the potential for serious type I error inflation of Cochran’s *Q* statistic when using standard first-order weights with weak instruments. In recent work, Verbanck *et al*.^22^ also noted this same tendency and proposed a simulation-based alternative to first-order weighting named ‘MR-PRESSO’. Our simulations show that modified weights can deliver much more reliable tests for heterogeneity than either first- or second-order weighting, and offer a simple alternative to MR-PRESSO.

Modified weights were also shown to be a more reliable tool for the detection and removal of outliers in a given data set, as apposed to first-order weights (which may detect too many outliers) and second-order weights (that may detect too few). Our simulations suggest that the exact weights should be used when testing for the overall presence of heterogeneity (referred to as the ‘global’ test by Verbanck *et al*.^22^) but that iterative weights are preferable if looking at the individual outliers. We suspect this is because exact weighting makes a more aggressive correction for regression dilution bias than iterative weighting. Its resulting estimate then makes more variants appear as outliers, because their ratio estimates are further away from the corrected slope. In effect, exact weighting leads to the detection of SNPs that are weak or pleiotropic.

An exciting finding of this paper is that the exact weighting also yields causal estimates that are remarkably robust to weak instrument bias. This opens up the potential for the significance threshold used to select SNPs as instruments to be set at a less stringent level. For example, in a specific analysis, there might be four SNPs that are associated with the exposure with a *p*-value less than 5 × 10^–^^8^ (which equates to an *F*-statistic of approximately 30 and above), but a total of 50 SNPs available that are associated with the exposure with a *p*-value less than 5 × 10^–^^6^ (which equates to an *F*-statistic of approximately 20 and above). Modified weights would then be potentially preferable as a tool to more effectively utilize this larger set of SNPs within an MR analysis.

There are two downsides to the use of exact weights with weak instruments. First, it can produce causal estimates with a reduced precision compared with simple first-order weighting (although this difference disappears as the number of instruments increases). Second, if weak instruments are ‘discovered’ and analysed using the same data, then SNP–exposure estimates are more susceptible to the ‘winner’s curse’ than strong instruments. In preliminary work conducted in tandem with this paper, Zhao *et al*.^14^ investigate the use of exact weighting for causal estimation and attempt to address both these issues. Specifically, they incorporate a penalized weight function within the exact weights. This reduces the effect of outliers (as apposed to explicit outlier removal) and increases the precision of the causal estimate. Sampling splitting is proposed to remove the effect of winner’s curse. The methods laid out in this paper differ from that of Zhao *et al*.^14^ in four important ways. First, we focus on the case of a multiplicative random-effects pleiotropy commonly used in summary-data MR, whereas Zhao *et al*. assume an additive random-effects model. Second, Zhao *et al*. derive and implement their method using profile-likelihood theory, whereas our approach is motivated and implemented using Cochran’s *Q* statistic. Third, we propose two forms of modified weighting (iterative and exact). Fourth, we describe how both iterative and exact weighting can be used to test for heterogeneity as well as for causal estimation. For further details on the link between our work and that of Zhao *et al*.,^14^ see Supplementary Appendix 1 (available as Supplementary data at *IJE* online).

## Limitations

Our conclusions regarding the use of modified weights are limited to the two-sample summary setting where SNP–outcome and SNP–exposure associations are estimated in independent but homogeneous samples. Further research would be required to extend modified weights to settings where there is partial overlap between samples or in the single-sample (total overlap) setting.

When Cochran’s *Q* statistic detects significant amounts of heterogeneity, it is prudent to test whether it is meaningfully biasing the analysis. This would indeed be the case if the heterogeneity were caused in part by directional pleiotropy with a non-zero mean. This would lead to bias in the IVW estimate, unless of course it was caused by a small number of SNPs that could be identified and removed from the analysis. MR-Egger regression^6^^,^^7^ could instead be used to address this. This approach simply regresses SNP–outcome associations on the SNP–exposure associations, tests for bias via its intercept and estimates a bias-adjusted causal effect via its slope. Observed heterogeneity around the MR-Egger fit can then be quantified using an extended version of Cochran’s *Q* statistic, Rücker’s $Q^{\prime }$,^7^^,^^23^ and each variant’s contribution to $Q^{\prime }$ can be used as the basis for outlier detection. Currently, MR-Egger and Rücker’s $Q^{\prime }$ statistic use first-order weights. Preliminary work suggests that modified weighting can be applied to MR-Egger regression to improve its performance—in terms of both causal-effect estimation and heterogeneity quantification—just as for an IVW analysis, but further development and validation of this method is required.

Software to implement all of the methods introduced in this paper can be found within the RadialMR package to perform two-sample summary-data MR, which can be downloaded from https://github.com/WSpiller/RadialMR.

**Box 1. Standard two-sample summary-data MR**

**The IV assumptions:** The canonical approach to MR assumes that a group of SNPs are valid IVs for the purposes of inferring the causal effect of an exposure, *X*, on an outcome, *Y*. That is, they are: associated with *X* (IV1); not associated with any confounders of *X* and *Y* (IV2); and can only be associated with *Y* through *X* (IV3). The IV assumptions are represented by the solid lines in the causal diagram below for a SNP *G_j_*, with unobserved confounding represented by *U*. Dotted lines represent dependencies between *G* and *U*, and *G* and *Y* that are prohibited by the IV assumptions. The causal effect of a unit increase in *X* on the outcome *Y*, denoted by *β*, is the quantity we are aiming to estimate.
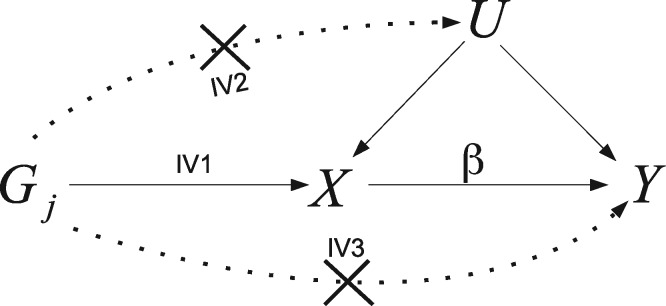

**The ratio estimate:** Assume that exposure *X* causally affects outcome *Y* linearly across all values of *X*, so that a hypothetical intervention that induced a 1-unit increase in *X* would induce a *β* increase in *Y*. Suppose also that all *L* SNPs predict the exposure via an additive linear model with no interactions. If SNP *j* is a valid IV, and the two study samples are homogeneous, then the underlying SNP–outcome association from Sample 1, Γ_*j*_, should be a scalar multiple of the underlying SNP–exposure association estimate from Sample 2, *γ_j_*, the scalar multiple being the causal effect *β*. That is:

$$\;\Gamma_{j}=\beta \gamma_{j}.$$

The ratio estimate for the causal effect of *X* on *Y* using SNP *j* (out of *L*), ${\hat{\beta }}_{j}={\hat{\Gamma }}_{j}/{\hat{\gamma }}_{j}$, where ${\hat{\Gamma }}_{j}$ is the estimate for SNP *j’*s association with the outcome (with standard error *σ_Y j_*) and ${\hat{\gamma }}_{j}$ is the estimate for SNP *j’*s association with the exposure (with standard error *σ_Xj_*).

**The IVW estimate:** The overall inverse-variance weighted (IVW) estimate for the causal effect obtained across *L* uncorrelated SNPs is then given by

$${\hat{\beta }}_{IVW}=\frac{\sum_{j=1}^{L}w_{j}{\hat{\beta }}_{j}}{\sum_{j=1}^{L}w_{j}},$$

where *w_j_* is the inverse-variance of ${\hat{\beta }}_{j}$. Cochran’s *Q* statistic:

(1) $$Q=\sum_{j=1}^{L}Q_{j}=\sum_{j=1}^{L}w_{j}{\left({\hat{\beta }}_{j}-{\hat{\beta }}_{IVW}\right)}^{2},$$

can then be used to test for the presence of heterogeneity. If heterogeneity is detected, this provides evidence of horizontal pleiotropy. Two popular choices for the inverse-variance weights used to calculate the IVW estimate and Cochran’s *Q* statistic are:

$$1\mathrm{st}\;\text{order}\;(\text{fixed}\;\text{effect})\;\text{weights}:\;\;w_{j}=\frac{{\hat{\gamma }}_{j}^{2}}{\sigma_{\text{Yj}}^{2}}$$

$$2\text{nd}\;\text{order}\;(\text{fixed}\;\text{effect})\;\text{weights}:\;\;w_{j}={\left(\frac{\sigma_{\text{Yj}}^{2}}{{\hat{\gamma }}_{j}^{2}}+\frac{{\hat{\Gamma }}_{j}^{2}\sigma_{\text{Xj}}^{2}}{{\hat{\gamma }}_{j}^{4}}\right)}^{-1}$$

In the two-sample setting, second-order weights are simplified because it is not necessary to include terms involving the covariance of ${\hat{\gamma }}_{j}$ and ${\hat{\Gamma }}_{j}$, since they are obtained from independent samples. For a more detailed description of the assumptions required by two-sample summary-data MR, see Bowden *et al*. [ 7^ ^].

**Box 2. Accounting for weak instruments under a fixed-effect model and testing for pleiotropy**

We start by writing down two models: first, the underlying data-generating model for the SNP–outcome association estimates under the assumption of no pleiotropy, which is a function of the causal effect and the true SNP–exposure association; and, second, the model that we actually *fit* to the data, which is a function of the causal effect and the SNP–exposure association *estimate*:

(2) $$\text{Underlying}\;\text{model}:\;\;{\hat{\Gamma }}_{j}=\beta \gamma_{j}+\sigma_{\text{Yj}}\epsilon_{j},\;\epsilon_{j}∼N(0,1)$$

(3) $$\text{Fitted}\;\text{model}\;\text{given}\;(2):\;{\hat{\Gamma }}_{j}=\beta {\hat{\gamma }}_{j}+\sqrt{\beta^{2}\sigma_{\text{Xj}}^{2}+\sigma_{\text{Yj}}^{2}}\epsilon_{j}^{\prime },\;\epsilon_{j}^{\prime }∼N(0,1).$$

Note that the variance of the error term in the fitted model has been inflated by a factor of $\beta^{2}\sigma_{Xj}^{2}$ by virtue of replacing *γ_j_* with its estimate in Equation (3). Dividing both sides of the fitted model by ${\hat{\gamma }}_{j}$, we can obtain a model for the *j*th ratio estimate, and from that an expression for its variance:

(4) $${\hat{\beta }}_{j}=\beta +\sqrt{\frac{\beta^{2}\sigma_{Xj}^{2}+\sigma_{Yj}^{2}}{{\hat{\gamma }}_{j}^{2}}}\;\epsilon_{j}^{\prime }\;\Rightarrow Var({\hat{\beta }}_{j})=\frac{\beta^{2}\sigma_{Xj}^{2}+\sigma_{Yj}^{2}}{{\hat{\gamma }}_{j}^{2}}.$$

The variance term $Var({\hat{\beta }}_{j})$ in Equation (4) is a function of the true causal effect *β*. Let its reciprocal inverse-variance weight be denoted as $w_{j}(\beta )=1/Var({\hat{\beta }}_{j})$. Using this weight, we now define the following modfied *Q* statistic and IVW estimate:

(5) $$Q_{m}(w(\beta ),\beta )=\sum_{j=1}^{L}w_{j}(\beta ){\left({\hat{\beta }}_{j}-\beta \right)}^{2},$$

(6) $${\hat{\beta }}_{IVW}=\frac{\sum_{j=1}^{L}w_{j}(\beta ){\hat{\beta }}_{j}}{\sum_{j=1}^{L}w_{j}(\beta )}.$$

The IVW estimate using first-order weights is obtained by replacing $w_{j}(\beta )$ with $w_{j}(0)$ in Equation (6). Likewise, its associated heterogeneity statistic is $Q_{m}(w(0),\beta )$. The IVW estimate using second-order weights is obtained by replacing $w_{j}(\beta )$ with $w_{j}({\hat{\beta }}_{j})$ in Equation (6). Likewise, its associated heterogeneity statistic is $Q_{m}(w({\hat{\beta }}_{j}),\beta )$.

We now introduce two new fixed-effect IVW estimates (and associated heterogeneity statistics) obtained via different weighting schemes.

**The ‘iterative’ IVW estimate**

Briefly, let ${\hat{\beta }}_{IVW(0)}$ be the IVW estimate obtained using first-order weights. Now define ${\hat{\beta }}_{IVW(1)}$ as the IVW estimate obtained from plugging $w_{j}({\hat{\beta }}_{IVW(0)})$ into Equation (6). Lastly, define ${\hat{\beta }}_{IVW(i)}$ as the IVW estimate obtained from plugging $w_{j}({\hat{\beta }}_{IVW(i-1)})$ into Equation (6). We call ${\hat{\beta }}_{IVW(i)}$ the *i*th ‘iterative’ IVW estimate and $Q_{m}(w({\hat{\beta }}_{IVW(i)}),\beta )$ its associated heterogeneity statistic. This iterative procedure should be repeated until the IVW estimate is stable.

**The ‘exact’ IVW estimate**

Although we obtain the first-order, second-order and iterative IVW estimates directly from Equation (6), each one has the property that it minimizes its equivalent *Q* statistic in Equation (5). Crucially, the weights of these *Q* statistics do not depend on *β* because a value (or estimate) has been substituted in its place.

In contrast, the exact IVW estimate is the value obtained from directly minimizing the generalized *Q* statistic $Q_{m}(w(\beta ),\beta )$ in Equation (5) with respect to *β*. Here, the weights are now allowed to be a proper function of *β* and affect the minimization. Letting ${\hat{\beta }}_{IVW}$ now represent the exact IVW estimate derived in this manner, $Q_{m}(w({\hat{\beta }}_{IVW}),{\hat{\beta }}_{IVW})$ is then its associated heterogeneity statistic.

**Box 3. Accounting for weak and pleiotropic instruments using exact weighting**

First define the following generalized *Q* statistic and weight function for the multiplicative random-effects model:

(7) $$\;Q(w(\beta ,\phi ),\beta )=\sum_{j=1}^{L}w_{j}(\beta ,\phi ){\left({\hat{\beta }}_{j}-\beta \right)}^{2},$$

(8) $$w_{j}(\beta ,\phi )={\left(\frac{\phi \sigma_{Yj}^{2}+\beta^{2}\sigma_{Xj}^{2}}{{\hat{\gamma }}_{j}^{2}}\right)}^{-1}.$$

Here, ϕ (which is greater than or equal to 1) is the multiplicative scale factor that quantifies the degree of heterogeneity.

**Inference for exact weighting under a fixed-effect model**

When ϕ is set to 1 in Equations (7) and (8), this is equivalent to assuming a fixed-effect model, and minimizing Equation (7) with respect to *β* gives the fixed-effect exact IVW estimate, as described in Box 2. We explore two ways to calculate the standard error of the fixed exact IVW estimate, denoted by ${\hat{\beta }}_{IVW}$. The first method uses the standard error formula:

(9) $$SE({\hat{\beta }}_{IVW})=\sqrt{\frac{1}{\sum_{j=1}^{L}w_{j}({\hat{\beta }}_{IVW},1)}},$$

to construct symmetric 95% confidence intervals for the causal effect as ${\hat{\beta }}_{IVW}\pm t_{.975,L-1}\times SE({\hat{\beta }}_{IVW1})$. Here, $t_{.975,L-1}$ is the 97.5th percentile of Student’s *t*-distribution with *L* – 1 degrees of freedom. This same procedure is used to derive confidence intervals for the IVW estimate under first-order, second-order and iterative weighting.

The second method directly inverts the *Q* statistic to find the confidence set:

(10) $$CI({\hat{\beta }}_{IVW},0.95)=\{\beta :Q(w(\beta ,1),\beta )\leq \chi_{L-1}^{2}(0.95)\},$$

where $\chi_{L-1}^{2}(0.95)$ is the 95th percentile of a chi-squared distribution with *L* – 1 degrees of freedom. In order to improve the properties of this approach with few instruments, we additionally replace the value 0.95 in Equation (10) with the value 2Φ(z)−1, where *z* is the 97.5th percentile of a *t*-distribution with *L* – 1 degrees of freedom and Φ() is the cumulative distribution function of a standard normal distribution. As *L* increases, 2Φ(z)−1 tends to 0.95 from above.

**Inference for exact weighting under a random-effects model**

The fixed-effect exact IVW estimate and its associated confidence intervals will only give reliable estimates if the fixed-effect model holds. In practice, substantial heterogeneity is generally present in MR studies, in which case a random-effects model should be adopted. The random-effects exact IVW estimate is obtained by finding the joint value of (*β*,ϕ) that solves:

(11) Q(w(β,ϕ),β)−(L−1)=0,

subject to the constraint that

(12) $$\frac{\partial Q(w(\beta ,\phi ),\beta )}{\partial \beta }=0.$$

It is not straightforward to obtain a reliable confidence interval for the causal parameter *β* using the inversion method—as in Equation 10—when over-dispersion is allowed. This is because it ignores uncertainty in the estimation of ϕ. Instead, we obtain an estimate for the variance of ${\hat{\beta }}_{IVW}$ using a standard non-parametric bootstrap algorithm. For further details, please see Supplementary Appendix 1 (available as Supplementary data at *IJE* online).

## Supplementary Material

dyy258_Supplementary_Material
