# Supplementary material for: Improving the accuracy of two-sample summary-data Mendelian randomization: moving beyond the NOME assumption
Source: Int J Epidemiol. 2018 Dec 18;48(3):728–42. doi: 10.1093/ije/dyy258 (PMC6659376; doi:10.1093/ije/dyy258)
Supplement: dyy258_Supplementary_Material [file dyy258_supplementary_material.pdf]

Supplementary material for: Improving the accuracy of two-sample summary data Mendelian randomization: moving beyond the NOME assumption

Jack Bowden[1,2*], Fabiola Del Greco M[3], Cosetta Minelli[4], Qingyuan Zhao[5], Debbie A Lawlor[1,2], Nuala A Sheehan[6], John Thompson[6] & George Davey Smith[1,2].

[1]*MRC Integrative Epidemiology Unit at the University of Bristol, U.K.*
[2]*Population Health Sciences, University of Bristol, U.K.*
[3]*Institute for Biomedicine, Eurac Research, Bolzano, Italy*
[4]*Population Health and Occupational Disease, NHLI, Imperial College, London, U.K.*
[5]*Department of Statistics, The Wharton School, University of Pennsylvania, U.S.A.*
[6]*Department of Health Sciences, University of Leicester, Leicester, U.K.*

*Address for correspondence:
Jack Bowden
MRC Integrative Epidemiology Unit
Oakfield House, Bristol, BS8 2BN, U.K
jack.bowden@bristol.ac.uk.

Appendix 1: Estimation and inference for fixed and random effects models

Let α_j represent the pleiotropic effect of SNP j on the outcome not via the exposure and let μ_α and σ^2_α denote the sample mean and variance, respectively, of all L pleiotropic effects. Suppose that the pleiotropic effects collectively satisfy the InSIDE assumption, and that the mean pleiotropic effect $\mu_\alpha = 0$. This is referred to as 'balanced' pleiotropy, and will induce heterogeneity amongst the ratio estimates. If heterogeneity is detected, inferences about the causal effect need to be adjusted to take this additional uncertainty into account, by assuming either by an additive random effects model [1] or a multiplicative random effects model [2]:

$$
\begin{aligned}
\text{Additive pleiotropy model: } \hat{\Gamma}_j &= \beta\gamma_j + \sqrt{\sigma^2_\alpha + \sigma^2_{Yj}}\epsilon_j && (1) \\
\text{Multiplicative pleiotropy model: } \hat{\Gamma}_j &= \beta\gamma_j + \sqrt{1+\sigma^2_\alpha}\sigma_{Yj}\epsilon_j. \\
&= \beta\gamma_j + \phi^{\frac{1}{2}}\sigma_{Yj}\epsilon_j. && (2)
\end{aligned}
$$

In line with current practice in two sample summary data Mendelian randomization [3, 4], we will focus predominantly on the multiplicative model 2, because it is automatically fitted by regression software. Specifically, we describe how to obtain IVW estimates (with associated standard errors and confidence intervals) and Q statistics using 1st order, 2nd order, iterative and exact weights. We will then conclude by discussing how our methods can be easily adapted for the additive random effects case. Note that, when there is no heterogeneity due to pleiotropy, $\sigma^2_\alpha = \alpha_j = 0$, ϕ=1 and (1) equals (2) (i.e. the fixed effect model).

The multiplicative case

First define the following generalized Q statistic for the multiplicative random effects model:

$$
\begin{aligned}
Q(w(\beta,\phi),\beta) &= \sum_{j=1}^{L} w_j(\beta,\phi)(\hat{\beta}_j - \beta)^2, && (3) \\
w_j(\beta,\phi) &= \left(\frac{\phi\sigma^2_{Yj} + \beta^2\sigma^2_{Xj}}{\hat{\gamma}^2_j}\right)^{-1} && (4)
\end{aligned}
$$

1st order, 2nd order and iterative weights

The 1st order IVW estimate $\hat{\beta}_{IVW}$ under a fixed effect model is equivalent to solving:

$$\frac{\partial Q(w(0,1),\beta)}{\partial\beta} = 0 \qquad (5)$$

Although β is set to zero in the above weight formula, this is actually a bi-product of 1st order weights making the NOME assumption (σ^2_{Xj}=0). This yields the standard analytic solution

$$\hat{\beta}_{IVW} = \frac{\sum_{j=1}^{L} w_j(0,1)\hat{\beta}_j}{\sum_{j=1}^{L} w_j(0,1)}. \tag{6}$$

The over-dispersion parameter is then calculated as

$$\hat{\phi} = \frac{Q(w(0,1), \hat{\beta}_{IVW})}{L-1}, \tag{7}$$

That is, the ratio of the 1st order Q statistic and its degrees of freedom under the null hypothesis of no over-dispersion (ϕ=1). A general formula for the standard error for $\hat{\beta}_{IVW1}$ is given by

$$SE(\hat{\beta}_{IVW1}) = \sqrt{\frac{\hat{\phi}}{\sum_{j=1}^{L} w_j(0,1)}} \tag{8}$$

Under a fixed effect model, the standard error is calculated by fixing $\hat{\phi}$ to 1 in equation 8. Under a multiplicative random effects model we fix $\hat{\phi}$ to be the maximum of 1 and its estimated value in 7, to ensure that the random effects IVW estimate is no more precise than the fixed effect estimate. Symmetric ninety-five percent confidence intervals for the causal effect are obtained as $\hat{\beta}_{IVW} \pm t_{.975,L-1} \times SE(\hat{\beta}_{IVW1})$ where $t_{.975,L-1}$ is the 97.5th percentile of student's t-distribution with $L-1$ degrees of freedom.

In order to obtain the IVW estimate using 2nd order weights (as well as Q statistics and standard errors under fixed and multiplicative random effects models) we replace $w_j(0,1)$ with $w_j(\hat{\beta}_j, 1)$ in equations 5, 6, 7 and 8. That is, the only difference between the 1st order and 2nd order IVW estimate is that we are replacing the guess β=0 with $\beta=\hat{\beta}_j$ in the weight function.

In order to obtain the ith iterative IVW estimate (as well as Q statistics and standard errors under fixed and random effects models) we replace $w_j(0,1)$ with $w_j(\hat{\beta}_{IVW(i-1)}, 1)$ in equations 5, 6, 7 and 8, where

- $\hat{\beta}_{IVW(i-1)}$ is the IVW estimate obtained from the ith iteration;
- $\hat{\beta}_{IVW(0)}$ is the IVW estimate obtained using 1st order weights.

That is, the only difference between the 1st order IVW estimate and the ith iterative IVW estimate is that we are replacing the guess β=0 with $\beta=\hat{\beta}_{IVW(i-1)}$ in the weight function.

Exact weights

Just as for 1st or 2nd order weighting, our method for calculating $\hat{\beta}_{IVW}$ using iterative weights preserves the property that the estimate remained the same under either a fixed or multiplicative random effects model. This also means that it produces IVW estimates and Q statistics that closely mirror those obtained under 1st order weighting with strong instruments, but leads to improvements in performance in the presence of weak instruments whilst being stochastically similar. Exact weights produce IVW estimates that can be very different with weak instruments, because a much more aggressive bias correction is enacted. Furthermore, the IVW estimate it produces is different under a fixed and multiplicative random effects model.

Fixed effect model: $\phi = 1$

The exact IVW estimate under a fixed effect model (ϕ=1), is the value of β that solves (or minimises):

$$\frac{\partial Q(w(\beta, 1), \beta)}{\partial \beta} = 0 \tag{9}$$

Note the difference between equation 9 and 5 is that the weight given to the jth contribution is an explicit function of β, and therefore directly affects the minimisation. We solve 9 using numerical methods. Plugging in the estimate obtained from (9) into the Q statistic, $Q(w(\hat{\beta}_{IVW}, 1), \hat{\beta}_{IVW})$ then provides an exact test of the null hypothesis of no heterogeneity. An estimate for the standard error of $\hat{\beta}_{IVW}$ can be obtained by plugging $w_j(\hat{\beta}_{IVW}, 1)$ into equation (8) with $\hat{\phi}$ set to 1 in order to obtain p-values and symmetric confidence intervals as above. Alternatively, a 95% confidence interval for $\hat{\beta}_{IVW}$ can be obtained directly by inverting the Q statistic to find the set:

$$CI(\hat{\beta}_{IVW}, \delta) = \{\beta : Q(w(\beta, 1), \beta) \leq \chi^2_{L-1}(0.95)\} \tag{10}$$

Where $\chi^2_{L-1}(0.95)$ is the 95th percentile of a chi-squared distribution with $L - 1$ degrees of freedom. In order to improve the properties of this approach with few instruments, we additionally replace the value 0.95 in (10) with the value $2\Phi(z) - 1$, where z is the 97.5th percentile of a t-distribution with $L - 1$ degrees of freedom and $\Phi()$ is the cumulative distribution function of a standard normal distribution. As L increases, $2\Phi(z) - 1$ tends to 0.95 from above.

Random effects model: joint estimation of β and ϕ

Joint estimation of the causal parameter β and the scale parameter ϕ under our stated model is known to be a challenging problem. For example, Zhao et al. [5] show that when the additive random effects model (1) is assumed, the maximum profile-likelihood estimates for β σ^2_α are biased. Translating this to our multiplicative model, this means that $Q(w(\beta, \phi), \beta)$ is not minimised at the true value of (β, ϕ), because as $\phi \to \infty$, $Q(w(\beta, \phi), \beta) \to 0$. Zhao et al. [5] follow the approach of McCullagh and Tibrishani [6] by instead maximising an 'adjusted' profile likelihood to obtain

bias adjusted estimates for β and σ_α^2. In this paper we take a simpler but analogous approach, by finding the value of ϕ that solves (or minimises):

$$Q(w(\beta,\phi),\beta) - (L-1) = 0, \tag{11}$$

subject to the constraint that

$$\frac{\partial Q(w(\beta,\phi),\beta)}{\partial \beta} = 0. \tag{12}$$

Specifically, solving equations 11 and 12 is approximately equivalent to solving equations (4.2) and (4.3) in [5], when translated to the multiplicative random effects model framework. In practice, we find restricting the upper and lower bound for ϕ to be between its estimate when using 2nd order weights and its estimate when using 1st order weights leads to stable and reliable estimates. This is because 1st order and 2nd order weights systematically under- and over-estimate the true value of ϕ respectively.

Zhao et al. [5] derive an expression an expression for their asymptotic variance of the causal effect after maximisation of the adjusted profile score. The stability of this variance estimate is then improved by incorporating additional penalized regression methods to down-weight outliers. It is not possible to obtain a confidence interval for the causal parameter β using the inversion method - as in equation 10 - when over-dispersion is allowed. This is because it ignores uncertainty in the estimation of ϕ. Instead we obtain an estimate for the variance of $\hat{\beta}_{IVW}$ using a non-parametric bootstrap algorithm. An equivalent parametric bootstrap algorithm was found to perform poorly with weak instruments, which is the very scenario we want it to work well. This is because generating boostrapped SNP-exposure associations $\hat{\gamma}_j^*$ from a $N(\hat{\gamma}_j, \sigma_{Xj}^2)$ distribution means that they contain twice as much uncertainty as the original estimates. This in turn leads to stronger regression dilution bias and under-estimation of the variance of $\hat{\beta}_{IVW}$. Our non-parametric estimation and bootstrap variance algorithm generally works well, but works least well when three factors come together: (1) the causal null β=0 is true (2) when there are few genetic instruments and (3) when the instruments are extremely weak.

Suggested adaptation to an additive random effects model

Translating the methods described from a multiplicative to an additive random effects model is straightforward. First define the following generalized Q statistic under the additive random effects model (1):

$$Q(w^*(\beta,\sigma^2_\alpha),\beta) = \sum_{j=1}^{L} w^*_j(\beta,\sigma^2_\alpha)(\hat{\beta}_j-\beta)^2, \quad (13)$$

$$w^*_j(\beta,\sigma^2_\alpha) = \left(\frac{\sigma^2_{Yj}+\beta^2\sigma^2_{Xj}+\sigma^2_\alpha}{\hat{\gamma}^2_j}\right)^{-1} \quad (14)$$

Note that now the second argument of the weight function is σ^2_α instead of ϕ (importantly, under a fixed effect model $\sigma^2_\alpha = 0$ but ϕ=1).

Fixed effect model

Under a fixed effect model, point estimates, standard errors and confidence intervals for the 1st order, 2nd order and iterative and exact IVW estimate (and their associated Q statistics) are identical to that previously described.

Random effects model: 1st order, 2nd order and iterative weights

When Cochran's Q statistic detects heterogeneity and a random effects model is adopted, it is common practice to estimate the σ^2_α using the DerSimonian and Laird method of moments estimator [1], which we denote by $\hat{\sigma}^2_\alpha$. The random effects estimate is then

$$\hat{\beta}_{IVW} = \frac{\sum_{j=1}^{L} w^*_j(\beta,\hat{\sigma}^2_\alpha)\hat{\beta}_j}{\sum_{j=1}^{L} w^*_j(\beta,\hat{\sigma}^2_\alpha)}, \quad (15)$$

where $w^*_j(\beta,\sigma^2_\alpha)$ equals: $w^*_j(0,\hat{\sigma}^2_\alpha)$ with 1st order weighting; $w^*_j(\hat{\beta}_j,\hat{\sigma}^2_\alpha)$ with 2nd order weighting; $w^*_j(\hat{\beta}_{IVW(i)},\hat{\sigma}^2_\alpha)$ for the (i+1)th iteration modified 2nd order weights. The standard error of the random effects estimate in each case is then

$$\sqrt{\frac{1}{\sum_{j=1}^{L} w^*_j(u,\hat{\sigma}^2_\alpha)}} \quad (16)$$

where u is replaced with 0, $\hat{\beta}_j$ and $\hat{\beta}_{IVW(i)}$ respectively.

Random effects model: Exact modified 2nd order weights

In this case we solve a near-identical pair of equations to the multiplicative case, namely

$$Q(w^*(\beta,\sigma^2_\alpha),\beta) - (L-1) = 0, \quad (17)$$

subject to the constraint that

$$\frac{\partial Q(w^*(\beta, \sigma_\alpha^2), \beta)}{\partial \beta} = 0, \tag{18}$$

and where σ_α^2 is constrained to lie between the DerSimonian and Laird estimate obtained using 1st and 2nd order weighting.

Appendix 2: Simulating summary data with no heterogeneity due to pleiotropy

Two-sample summary data MR studies comprising L SNP-exposure and SNP outcome association estimates $(\hat{\Gamma}_j, \hat{\gamma}_j)$ were generated from the following normal models:

$$\hat{\gamma}_j \sim N(\gamma_j, \sigma^2_{Xj}), \quad \hat{\Gamma}_j \sim N(\beta\gamma_j, \sigma^2_{Yj}) \tag{19}$$

given parameter vector values for $(\gamma_j, \sigma^2_{Xj}, \sigma^2_{Yj})$ and the causal parameter β. Under these models, the F-statistic for SNP j can be approximated by $\hat{\gamma}_j^2/\sigma^2_{Xj}$. Data generated under model (19) furnishes a set of ratio estimates between which no additional variation should exist as their F-statistics grows large (because NOME is satisfied), or if the causal effect (β) equals zero. To highlight this the γ_j parameters were simulated from a Uniform(0.34,1.1) distribution and σ_{Xj} was simulated from a Uniform(0.06,UB) distribution. By varying UB between 0.095 and 1 we were able to mimic MR studies with weak instruments (a mean F-statistic of 10) and strong instruments (a mean F-statistic of 100). Data were simulated for a range of causal effects and, across all scenarios, σ_{Yj} was simulated from a Uniform(0.015,0.11) distribution.

Testing for heterogeneity under no pleiotropy: L=10 and 100 variants

Mean	1st order w_j		2nd order w_j		Modified w_j			
					Iterative		Exact	
F	Q	T1E(Q)	Q	T1E(Q)	Q	T1E(Q)	Q	T1E(Q)
				**No heterogeneity, β=0**				
100	9.1	0.055	8.7	0.038	9.1	0.054	9.1	0.054
61	9.0	0.051	8.2	0.023	9.0	0.050	9.0	0.050
40	9.0	0.051	7.7	0.013	9.0	0.050	9.0	0.050
25	9.0	0.049	6.6	0.003	8.9	0.046	8.9	0.046
10	8.9	0.050	4.5	0.000	8.6	0.043	8.4	0.038
				**No heterogeneity, β=0.05**				
100	9.1	0.052	8.6	0.036	9.0	0.050	9.0	0.050
61	9.2	0.054	8.2	0.023	9.0	0.048	9.0	0.048
40	9.3	0.061	7.6	0.015	9.0	0.053	9.0	0.052
25	9.7	0.073	6.6	0.006	9.0	0.051	8.9	0.048
10	11.7	0.167	4.9	0.001	9.6	0.082	8.9	0.052
				**No heterogeneity, β=0.1**				
100	9.2	0.058	8.5	0.032	9.0	0.050	9.0	0.050
61	9.6	0.070	8.2	0.023	9.0	0.049	9.0	0.048
40	10.1	0.090	7.6	0.014	9.0	0.049	8.9	0.047
25	11.8	0.165	6.8	0.007	9.2	0.055	9.0	0.046
10	19.5	0.480	5.6	0.008	10.9	0.134	8.9	0.048

Table 1: *Mean Q statistic and type I error rate (T1E) of 1st order, 2nd order, iterative and exact weights. Results calculated over 10,000 simulated data sets of L=10 variants. Type I error rate (T1E(Q)) refers to the proportion of times Q is greater than the upper 95th percentile of a χ^2_9 distribution.*

Mean	1st order w_j		2nd order w_j		Modified w_j			
					Iterative		Exact	
F	Q	T1E(Q)	Q	T1E(Q)	Q	T1E(Q)	Q	T1E(Q)
				**No heterogeneity, β=0**				
100	99.0	0.050	94.4	0.017	99.0	0.050	99.0	0.050
61	98.9	0.050	90.3	0.007	98.9	0.050	98.9	0.050
40	98.9	0.049	83.9	0.001	98.8	0.049	98.8	0.049
25	99.0	0.050	72.9	0.000	98.9	0.049	98.9	0.049
10	99.0	0.047	50.8	0.000	98.6	0.045	98.4	0.043
				**No heterogeneity, β=0.05**				
100	99.9	0.059	94.4	0.019	99.0	0.053	99.0	0.053
61	100.6	0.064	90.2	0.006	98.9	0.048	98.8	0.048
40	102.8	0.087	84.4	0.001	99.3	0.055	99.2	0.054
25	107.1	0.148	73.9	0.000	99.4	0.055	98.8	0.048
10	130.9	0.629	56.4	0.000	105.7	0.135	98.6	0.048
				**No heterogeneity, β=0.1**				
100	102.6	0.081	94.4	0.019	99.1	0.046	99.0	0.045
62	105.8	0.127	90.2	0.008	98.8	0.048	98.6	0.047
40	113.2	0.265	84.9	0.002	99.3	0.053	98.9	0.049
25	132.5	0.651	76.6	0.000	100.9	0.069	99.3	0.052
10	227.5	0.999	67.9	0.001	112.3	0.261	98.8	0.045

Table 2: *Mean Q statistic and type I error rate (T1E) of 1st order, 2nd order, iterative and exact weights. Results calculated over 10,000 simulated data sets of L=100 variants. Type I error rate (T1E(Q)) refers to the proportion of times Q is greater than the upper 95th percentile of a* χ^2_{99} *distribution.*

Appendix 3: Power to detect heterogeneity under an additive random effects model

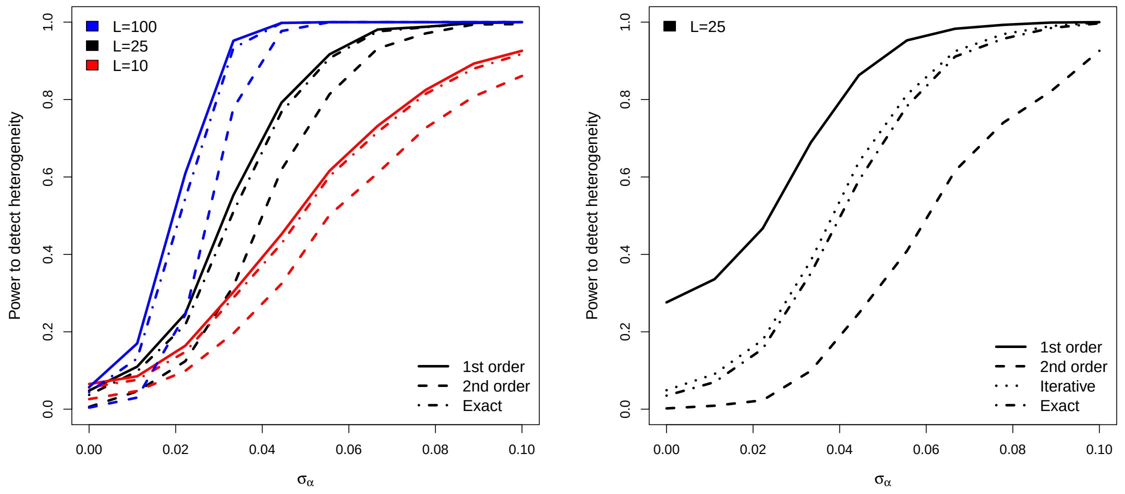

Figure 1: *Power of Cochran's Q statistic to detect heterogeneity as a function of the pleiotropy standard deviation (σ_α) and number of SNPs (L) using 1st order, 2nd order iterative and exact weights under an additive pleiotropy model.*

Appendix 4: Detecting outliers using individual components of Q

10,000 summary associations are simulated for 25 SNPs for a range of mean F-statistics, a causal effect of 0.05 and under the assumption of no heterogeneity due to pleiotropy. That is, just as for rows 6-10 in Table 1 of the main paper. Each data set of 25 SNPs is then augmented with a single outlying SNP (with a fixed pleiotropic effect) which almost triples the magnitude of the observed heterogeneity across all 26 SNPs, as measured by Cochran's Q. Table 3 shows, for each weighting scheme: the mean Q statistic, the median and mean number of 'outliers' detected at the 5% level and the proportion of times that the true outlier is detected (P^*) as F is varied from 100 to 10. Figure 2 shows equivalent box plots of the outlier data, to highlight further summary quantities such as the inter-quartile range.

We would expect approximately 25×0.05 = 1.25 of the normal, non-heterogeneous SNPs to be declared outliers by chance at the 5% significance level, and hope that the true outlier is detected as often as possible, giving an ideal mean total of 2.25. As the mean F-statistic decreases, the total number of outliers detected using 1st order weights steadily increases beyond this value (although the probability of detecting the true outlier stays constant at ≈ 95%). By contrast, the total number of outliers detected using 2nd order weights substantially decreases, as well as the ability to detect the true outlier. For example, when F is 10, the true outlier is detected in less than 30% of cases. The performance of modified 2nd order weights is much more stable across the range of instrument strengths, with the median and mean number of outliers never increasing beyond 2 and 3 respectively. However, in this case it is

the iterative rather than the exact weights that appear to perform best. For example, when the mean F-statistic is 10 the power to detect the true outlier drops to only 87% using the exact approach, but stays at 94% for the iterative approach. Moreover, the box plots in Figure 2 show that the number of outliers detected across the simulations is much more variable for the exact, compared to the iterative implementation.

Mean		1st order w_j		2nd order w_j		Modified w_j		
						Iterative		Exact
		'Outliers' detected		'Outliers' detected		'Outliers' detected		'Outliers' detected
F	Q	(Median,Mean,P^*)	Q	(Median,Mean,P^*)	Q	(Median,Mean,P^*)	Q	(Median,Mean,P^*)
			**No heterogeneity for 25 SNPs + 1 outlier, β=0.05**					
100	67.6	(2,2.54,0.94)	42.3	(2,1.98,0.93)	64.1	(2,2.44,0.94)	63.4	(2,2.69,0.94)
61	68.5	(2,2.55,0.94)	37.0	(2,1.78,0.90)	62.8	(2,2.38,0.94)	61.4	(2,2.77,0.94)
40	69.6	(2,2.58,0.94)	31.6	(1,1.50,0.81)	60.4	(2,2.32,0.94)	57.9	(2,2.82,0.94)
24	71.2	(2,2.71,0.95)	25.8	(1,1.10,0.62)	56.8	(2,2.25,0.94)	52.6	(2,2.82,0.94)
10	80.0	(3,3.27,0.95)	17.5	(0,0.53,0.28)	53.2	(2,2.23,0.94)	41.1	(2,2.58,0.87)

Table 3: *The number of outliers detected at the* 5% *level by Cochran's Q statistic when using 1st order, 2nd order, iterative and exact weights for MR summary data containing 25 non-heterogeneous SNPs and 1 outlier.*

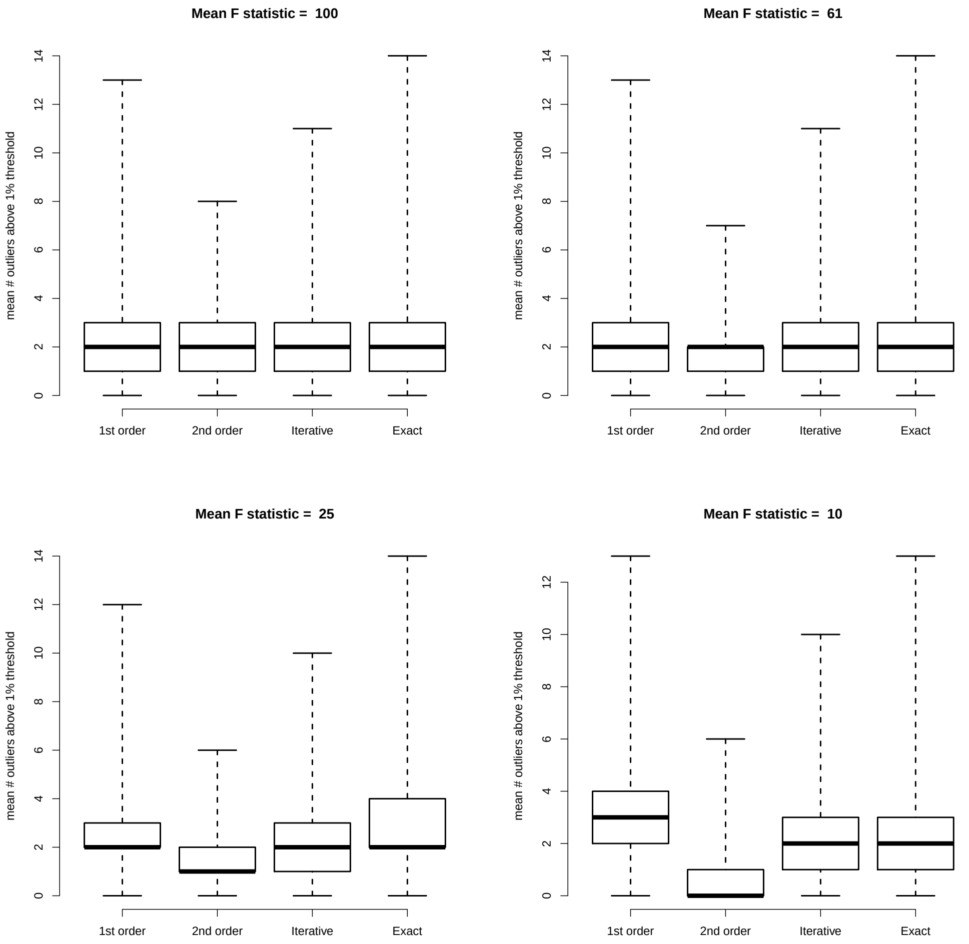

Figure 2: *Box plots summarising the total number of outliers detected by Cochran's Q statistic using 1st order, 2nd order, iterative and exact weights when the mean F-statistic is varied betwen 100 (top-left) and 10 (bottom-right). Each box shows the 1st quartile, median line and 3rd quartile, so that its height represent the inter-quartile range. Box 'whiskers' representing the full outlier range are also shown.*

Appendix 5: Estimator performance: Results for L=10 and 100 under a fixed effect model (no heterogeneity)

Mean	1st order w_j	2nd order w_j	Modified w_j		
			Iterative	Exact	
F	$\hat{\beta}_{IVW}$(SE); CF	$\hat{\beta}_{IVW}$(SE); CF	$\hat{\beta}_{IVW}$(SE); CF	$\hat{\beta}_{IVW}$(SE);CF$_1$	CF$_2$
			**No Heterogeneity, β=0**		
100	0.000 (0.018); 0.948	0.000 (0.018); 0.950	0.000 (0.018); 0.949	0.000 (0.019); 0.976	0.957
62	0.000 (0.018); 0.948	0.000 (0.018); 0.949	0.000 (0.018); 0.948	0.000 (0.019); 0.974	0.962
40	0.000 (0.018); 0.951	0.000 (0.017); 0.948	0.000 (0.018); 0.952	0.000 (0.019); 0.974	0.963
25	0.000 (0.018); 0.947	0.000 (0.016); 0.941	0.000 (0.018); 0.951	0.000 (0.019); 0.968	0.967
10	0.000 (0.016); 0.947	0.000 (0.012); 0.924	0.000 (0.016); 0.954	0.000 (0.019); 0.892	0.982
			**No Heterogeneity, β=0.05**		
100	0.050 (0.018); 0.949	0.049 (0.018); 0.951	0.050 (0.019); 0.951	0.050 (0.019); 0.973	0.956
61	0.049 (0.018); 0.947	0.048 (0.018); 0.947	0.049 (0.019); 0.950	0.050 (0.019); 0.975	0.963
40	0.049 (0.018); 0.947	0.046 (0.018); 0.942	0.049 (0.019); 0.952	0.051 (0.019); 0.972	0.963
25	0.046 (0.019); 0.936	0.042 (0.017); 0.904	0.047 (0.019); 0.942	0.051 (0.020); 0.963	0.970
10	0.034 (0.018); 0.801	0.028 (0.014); 0.615	0.035 (0.019); 0.829	0.048 (0.023); 0.860	0.976
			**No Heterogeneity, β=0.1**		
100	0.099 (0.019); 0.944	0.097 (0.019); 0.945	0.099 (0.019); 0.948	0.100 (0.020); 0.977	0.962
61	0.099 (0.019); 0.935	0.095 (0.019); 0.938	0.099 (0.020); 0.947	0.101 (0.020); 0.972	0.962
40	0.097 (0.019); 0.931	0.091 (0.019); 0.919	0.097 (0.020); 0.945	0.101 (0.021); 0.972	0.969
25	0.093 (0.020); 0.902	0.084 (0.018); 0.840	0.093 (0.022); 0.932	0.102 (0.023); 0.961	0.972
10	0.068 (0.022); 0.662	0.058 (0.017); 0.418	0.072 (0.024); 0.755	0.103 (0.028); 0.904	0.980

Table 4: *Mean causal estimate $\hat{\beta}_{IVW}$, standard error (SE) and coverage frequency (CF, CF$_1$ and CF$_2$) of the 95% confidence interval when using 1st order, 2nd order iterative and exact weights. L=10*

Mean	1st order w_j	2nd order w_j	Modified w_j		
			Iterative	Exact	
F	$\hat{\beta}_{IVW}$(SE); CF	$\hat{\beta}_{IVW}$(SE); CF	$\hat{\beta}_{IVW}$(SE); CF	$\hat{\beta}_{IVW}$(SE);CF$_1$	CF$_2$
			**No Heterogeneity, β=0**		
100	0.000 (0.005); 0.950	0.000 (0.005); 0.950	0.000 (0.005); 0.950	0.000 (0.005); 0.950	0.948
61	0.000 (0.005); 0.949	0.000 (0.005); 0.949	0.000 (0.005); 0.949	0.000 (0.005); 0.948	0.947
40	0.000 (0.005); 0.947	0.000 (0.005); 0.945	0.000 (0.005); 0.948	0.000 (0.005); 0.942	0.947
25	0.000 (0.005); 0.951	0.000 (0.005); 0.941	0.000 (0.005); 0.952	0.000 (0.005); 0.928	0.946
10	0.000 (0.004); 0.948	0.000 (0.003); 0.930	0.000 (0.004); 0.952	0.000 (0.004); 0.781	0.950
			**No Heterogeneity, β=0.05**		
100	0.050 (0.005); 0.948	0.049 (0.005); 0.941	0.050 (0.005); 0.949	0.050 (0.005); 0.951	0.943
61	0.049 (0.005); 0.937	0.047 (0.005); 0.912	0.049 (0.006); 0.941	0.050 (0.006); 0.944	0.947
40	0.048 (0.005); 0.928	0.045 (0.005); 0.829	0.048 (0.006); 0.935	0.050 (0.006); 0.944	0.942
25	0.045 (0.005); 0.836	0.040 (0.005); 0.498	0.046 (0.006); 0.865	0.050 (0.006); 0.929	0.948
10	0.031 (0.005); 0.095	0.026 (0.004); 0.003	0.033 (0.005); 0.192	0.050 (0.006); 0.856	0.948
			**No Heterogeneity, β=0.1**		
100	0.099 (0.006); 0.940	0.097 (0.006); 0.916	0.099 (0.006); 0.947	0.100 (0.006); 0.950	0.951
61	0.098 (0.006); 0.916	0.094 (0.006); 0.819	0.098 (0.006); 0.934	0.100 (0.006); 0.948	0.949
40	0.096 (0.006); 0.856	0.090 (0.006); 0.575	0.096 (0.006); 0.895	0.100 (0.006); 0.946	0.947
25	0.090 (0.006); 0.612	0.082 (0.006); 0.141	0.092 (0.007); 0.749	0.100 (0.007); 0.936	0.945
10	0.063 (0.007); 0.006	0.053 (0.005); 0.000	0.072 (0.007); 0.052	0.101 (0.008); 0.889	0.960

Table 5: *Mean causal estimate $\hat{\beta}_{IVW}$, standard error (SE) and coverage frequency (CF, CF$_1$ and CF$_2$) of the 95% confidence interval when using 1st order, 2nd order, iterative and exact weights. L=100*

Appendix 6. Estimator performance: Results for L=10 and 100, multiplicative random effects model

Mean	1st order w_j	2nd order w_j	Modified w_j		
			Iterative	Exact	
F	$\hat{\beta}_{IVW}$(SE); CF	$\hat{\beta}_{IVW}$(SE); CF	$\hat{\beta}_{IVW}$(SE); CF	$\hat{\beta}_{IVW}$(SE);CF	$\hat{\phi}$
			**Heterogeneity, β=0**		
100	0.000 (0.026); 0.949	0.000 (0.025); 0.950	0.000 (0.026); 0.950	0.000 (0.027); 0.944	2.034
61	0.000 (0.026); 0.947	0.000 (0.024); 0.948	0.000 (0.026); 0.949	0.000 (0.028); 0.947	2.030
40	0.000 (0.026); 0.947	0.000 (0.024); 0.945	0.000 (0.026); 0.950	0.000 (0.029); 0.946	2.023
24	0.000 (0.025); 0.948	0.000 (0.022); 0.939	0.000 (0.025); 0.953	0.000 (0.034); 0.952	1.995
10	-0.001 (0.022); 0.951	0.000 (0.016); 0.922	-0.001 (0.023); 0.958	0.002 (0.162); 0.973	1.912
			**Heterogeneity, β=0.05**		
100	0.050 (0.026); 0.947	0.048 (0.025); 0.948	0.050 (0.026); 0.948	0.050 (0.027); 0.943	2.031
61	0.050 (0.026); 0.948	0.047 (0.025); 0.946	0.050 (0.026); 0.951	0.051 (0.028); 0.947	2.012
40	0.049 (0.026); 0.949	0.044 (0.024); 0.941	0.049 (0.026); 0.953	0.051 (0.029); 0.950	2.027
25	0.046 (0.026); 0.940	0.039 (0.022); 0.906	0.047 (0.027); 0.945	0.051 (0.035); 0.953	2.028
10	0.034 (0.024); 0.859	0.027 (0.018); 0.678	0.036 (0.025); 0.873	0.048 (0.163); 0.972	1.982
			**Heterogeneity, β=0.1**		
100	0.099 (0.026); 0.947	0.096 (0.026); 0.948	0.099 (0.027); 0.950	0.100 (0.028); 0.944	2.035
61	0.099 (0.026); 0.947	0.093 (0.026); 0.946	0.099 (0.027); 0.956	0.101 (0.028); 0.949	2.027
40	0.098 (0.026); 0.942	0.089 (0.025); 0.924	0.098 (0.028); 0.950	0.101 (0.030); 0.949	2.029
25	0.093 (0.027); 0.921	0.080 (0.024); 0.846	0.093 (0.029); 0.940	0.101 (0.037); 0.952	2.050
10	0.068 (0.027); 0.728	0.054 (0.020); 0.453	0.073 (0.030); 0.809	0.099 (0.134); 0.962	2.112

Table 6: *Mean causal estimate $\hat{\beta}_{IVW}$, standard error (SE) and coverage frequency (CF) of the 95% confidence interval when using 1st order, 2nd order, iterative and exact weights. L=10. $\hat{\phi}$ = variance inflation factor.*

Mean	1st order w_j	2nd order w_j	Modified w_j		
			Iterative	Exact	
F	$\hat{\beta}_{IVW}$(SE); CF	$\hat{\beta}_{IVW}$(SE); CF	$\hat{\beta}_{IVW}$(SE); CF	$\hat{\beta}_{IVW}$(SE);CF	$\hat{\phi}$
			**Heterogeneity, β=0**		
100	0.000 (0.008); 0.951	0.000 (0.007); 0.950	0.00 (0.008); 0.951	0.00 (0.008); 0.944	2.001
61	0.000 (0.008); 0.953	0.000 (0.007); 0.953	0.000 (0.008); 0.953	0.00 (0.008); 0.945	2.001
40	0.000 (0.008); 0.949	0.000 (0.007); 0.945	0.000 (0.008); 0.950	0.00 (0.008); 0.942	2.000
25	0.000 (0.007); 0.949	0.000 (0.006); 0.940	0.000 (0.007); 0.950	0.00 (0.008); 0.939	1.999
10	0.000 (0.006); 0.952	0.000 (0.004); 0.927	0.000 (0.006); 0.957	0.00 (0.011); 0.950	1.987
			**Heterogeneity, β=0.05**		
100	0.050 (0.008); 0.949	0.048 (0.007); 0.942	0.050 (0.008); 0.950	0.05 (0.008); 0.943	1.996
61	0.049 (0.008); 0.943	0.046 (0.007); 0.907	0.049 (0.008); 0.946	0.05 (0.008); 0.938	1.998
40	0.048 (0.008); 0.934	0.043 (0.007); 0.820	0.048 (0.008); 0.941	0.05 (0.008); 0.940	2.004
25	0.045 (0.007); 0.893	0.038 (0.007); 0.536	0.046 (0.008); 0.914	0.05 (0.008); 0.946	1.998
10	0.031 (0.007); 0.229	0.023 (0.005); 0.009	0.033 (0.007); 0.394	0.05 (0.011); 0.942	1.995
			**Heterogeneity, β=0.1**		
100	0.099 (0.008); 0.942	0.095 (0.008); 0.908	0.099 (0.008); 0.948	0.10 (0.008); 0.942	2.000
61	0.098 (0.008); 0.931	0.092 (0.008); 0.812	0.098 (0.008); 0.943	0.10 (0.008); 0.940	2.004
40	0.096 (0.008); 0.904	0.087 (0.008); 0.573	0.096 (0.008); 0.930	0.10 (0.008); 0.942	1.998
25	0.090 (0.008); 0.742	0.077 (0.007); 0.162	0.092 (0.009); 0.842	0.10 (0.009); 0.941	2.002
10	0.063 (0.008); 0.025	0.049 (0.006); 0.000	0.072 (0.009); 0.167	0.10 (0.013); 0.947	2.003

Table 7: *Mean causal estimate $\hat{\beta}_{IVW}$, standard error (SE) and coverage frequency (CF) of the 95% confidence interval when using 1st order, 2nd order, iterative and exact weights. L=100. $\hat{\phi}$ = variance inflation factor.*

Appendix 7: Power to detect a causal effect: L=10,25 and 100

Mean F	β	1st order w_j	2nd order w_j	Modified w_j Iterative	Exact
100	0.00	0.054	0.053	0.054	0.058
61	0.00	0.046	0.042	0.044	0.052
40	0.00	0.046	0.046	0.042	0.048
25	0.00	0.058	0.065	0.052	0.049
10	0.00	0.049	0.080	0.046	0.028
100	0.01	0.074	0.072	0.072	0.076
61	0.01	0.068	0.069	0.066	0.072
40	0.01	0.080	0.081	0.076	0.082
25	0.01	0.063	0.066	0.058	0.061
10	0.01	0.060	0.090	0.050	0.030
100	0.02	0.104	0.104	0.101	0.104
61	0.02	0.114	0.114	0.110	0.116
40	0.02	0.117	0.122	0.110	0.114
25	0.02	0.109	0.118	0.100	0.089
10	0.02	0.100	0.134	0.082	0.036
100	0.03	0.211	0.205	0.208	0.198
61	0.03	0.194	0.186	0.188	0.179
40	0.03	0.194	0.194	0.184	0.180
25	0.03	0.168	0.178	0.153	0.140
10	0.03	0.158	0.184	0.128	0.070
100	0.04	0.296	0.290	0.294	0.281
61	0.04	0.286	0.281	0.278	0.268
40	0.04	0.289	0.274	0.270	0.252
25	0.04	0.278	0.280	0.252	0.220
10	0.04	0.204	0.235	0.178	0.093
100	0.05	0.436	0.429	0.432	0.402
61	0.05	0.446	0.432	0.433	0.406
40	0.05	0.422	0.408	0.400	0.384
25	0.05	0.404	0.386	0.380	0.321
10	0.05	0.288	0.292	0.242	0.127

Table 8: *Power to detect a causal effect at the 5% significance level as a function of the causal parameter β and the mean instrument strength using 1st order, 2nd order, iterative and exact weights. L=10.*

Mean F	β	1st order w_j	2nd order w_j	Modified w_j Iterative	Modified w_j Exact
100	0.00	0.060	0.058	0.059	0.067
61	0.00	0.046	0.046	0.046	0.056
40	0.00	0.044	0.052	0.044	0.056
25	0.00	0.048	0.048	0.040	0.058
10	0.00	0.053	0.078	0.043	0.036
100	0.01	0.100	0.098	0.098	0.118
61	0.01	0.102	0.099	0.102	0.108
40	0.01	0.098	0.103	0.092	0.110
25	0.01	0.092	0.100	0.086	0.094
10	0.01	0.083	0.110	0.070	0.044
100	0.02	0.246	0.244	0.244	0.258
61	0.02	0.244	0.243	0.241	0.246
40	0.02	0.214	0.212	0.209	0.224
25	0.02	0.236	0.228	0.222	0.219
10	0.02	0.168	0.202	0.146	0.098
100	0.03	0.446	0.434	0.442	0.450
61	0.03	0.451	0.438	0.444	0.438
40	0.03	0.444	0.426	0.436	0.444
25	0.03	0.424	0.415	0.403	0.406
10	0.03	0.317	0.310	0.287	0.178
100	0.04	0.668	0.664	0.668	0.662
61	0.04	0.667	0.660	0.660	0.664
40	0.04	0.680	0.662	0.673	0.665
25	0.04	0.620	0.606	0.608	0.590
10	0.04	0.460	0.470	0.426	0.286
100	0.05	0.853	0.848	0.852	0.839
61	0.05	0.836	0.826	0.834	0.817
40	0.05	0.836	0.812	0.830	0.815
25	0.05	0.804	0.765	0.788	0.770
10	0.05	0.623	0.617	0.608	0.400

Table 9: *Power to detect a causal effect at the 5% significance level as a function of the causal parameter β and the mean instrument strength using 1st order, 2nd order, iterative and exact weights. L=25.*

Mean F	β	1st order w_j	2nd order w_j	Modified w_j	
				Iterative	Exact
100	0.00	0.054	0.056	0.054	0.060
61	0.00	0.048	0.045	0.048	0.046
40	0.00	0.044	0.056	0.045	0.060
25	0.00	0.048	0.058	0.046	0.054
10	0.00	0.044	0.068	0.040	0.044
100	0.01	0.268	0.262	0.267	0.278
61	0.01	0.250	0.252	0.250	0.270
40	0.01	0.228	0.224	0.227	0.248
25	0.01	0.238	0.230	0.238	0.238
10	0.01	0.173	0.198	0.158	0.176
100	0.02	0.740	0.738	0.740	0.738
61	0.02	0.705	0.704	0.704	0.709
40	0.02	0.703	0.688	0.702	0.702
25	0.02	0.676	0.654	0.672	0.679
10	0.02	0.522	0.509	0.502	0.496
100	0.03	0.967	0.965	0.966	0.964
61	0.03	0.960	0.956	0.960	0.958
40	0.03	0.957	0.950	0.956	0.952
25	0.03	0.943	0.929	0.940	0.938
10	0.03	0.844	0.811	0.840	0.808
100	0.04	1.000	1.000	1.000	1.000
61	0.04	0.998	0.998	0.998	0.998
40	0.04	0.997	0.996	0.997	0.996
25	0.04	0.999	0.998	1.000	0.998
10	0.04	0.968	0.950	0.966	0.950
100	0.05	1.000	1.000	1.000	1.000
61	0.05	1.000	1.000	1.000	1.000
40	0.05	1.000	1.000	1.000	1.000
25	0.05	1.000	1.000	1.000	1.000
10	0.05	0.992	0.990	0.996	0.992

Table 10: *Power to detect a causal effect at the 5% significance level as a function of the causal parameter β and the mean instrument strength using 1st order, 2nd order, iterative and exact weights. L=100.*

References

[1] DerSimonian R, Laird N. Meta-analysis in clinical trials. *Controlled Clinical Trials* 1986; **7**:177–188.

[2] Thompson SG, Sharp S. Explaining heterogeneity in meta-analysis: a comparison of methods. *Statistics in Medicine* 1999; **18**:2693–2708.

[3] Bowden J, Davey Smith G, Burgess S. Mendelian randomization with invalid instruments: effect estimation and bias detection through Egger regression. *International Journal of Epidemiology* 2015; **44**:512–525.

[4] Bowden J, Del Greco M F, Minelli C, Davey Smith G, Sheehan NA, Thompson JR. A framework for the investigation of pleiotropy in two-sample summary data Mendelian randomization *Statistics in Medicine* 2017; **36**: 1783–1802

[5] Zhao Q, Wang J, Hemani G, Bowden J, Small D. Statistical inference in two-sample summary data Mendelian randomization using a robust adjusted profile score. Technical report, University of Pennsylvania 2018. `http://www-stat.wharton.upenn.edu/~qyzhao/papers/mr_raps.pdf`

[6] McCullagh P, Tibshirani R. A simple method for the adjustment of profile likelihoods. Journal of the Royal Statistical Society. Series B 1990; **52**: 325–344.
